# Neopterin and CXCL-13 in Diagnosis and Follow-Up of *Trypanosoma brucei gambiense* Sleeping Sickness: Lessons from the Field in Angola

**DOI:** 10.1155/2019/6070176

**Published:** 2019-11-23

**Authors:** Julien Bonnet, Philippe Vignoles, Natalia Tiberti, Vatunga Gedeão, Alexandre Hainard, Natacha Turck, Theophile Josenando, Joseph M Ndung'u, Jean-Charles Sanchez, Bertrand Courtioux, Sylvie Bisser

**Affiliations:** ^1^Institute of Neuroepidemiology and Tropical Neurology, School of Medicine, CNRS FR 3503 GEIST, University of Limoges, INSERM UMR1094 Tropical Neuroepidemiology, Limoges, France; ^2^Translational Biomarker Group, Department of Human Protein Sciences, University of Geneva, Geneva, Switzerland; ^3^Department of Infectious—Tropical Diseases and Microbiology, IRCCS Sacro Cuore Don Calabria Hospital, Negrar, Verona, Italy; ^4^Instituto de Combate e Controlo das Tripanossomiases (ICCT), Luanda, Angola; ^5^Foundation for Innovative New Diagnostics (FIND), Geneva, Switzerland; ^6^Pasteur Institute in French Guiana, 23 Boulevard Pasteur, 973006 Cayenne Cedex, French Guiana

## Abstract

Human African Trypanosomiasis may become manageable in the next decade with fexinidazole. However, currently stage diagnosis remains difficult to implement in the field and requires a lumbar puncture. Our study of an Angolan cohort of *T. b. gambiense*-infected patients used other staging criteria than those recommended by the WHO. We compared WHO criteria (cell count and parasite identification in the CSF) with two biomarkers (neopterin and CXCL-13) which have proven potential to diagnose disease stage or relapse. Biological, clinical, and neurological data were analysed from a cohort of 83 patients. A neopterin concentration below 15.5 nmol/L in the CSF denoted patients with stage 1 disease, and a concentration above 60.31 nmol/L characterized patients with advanced stage 2 (trypanosomes in CSF and/or cytorachia higher than 20 cells) disease. CXCL-13 levels below 91.208 pg/mL denoted patients with stage 1 disease, and levels of CXCL-13 above 395.45 pg/mL denoted patients with advanced stage 2 disease. Values between these cut-offs may represent patients with intermediate stage disease. Our work supports the existence of an intermediate stage in HAT, and CXCL-13 and neopterin levels may help to characterize it.

## 1. Introduction

It is hoped that sleeping sickness will become manageable within the next decade, as suggested by the WHO [1, 2]. In 2016, the number of patients with sleeping sickness has been reported as fewer than 4,000 but there are still unreported cases, and the estimate of actual cases is around 20,000 infected people in the remaining endemic countries in Africa. The availability of easy-to-use molecules such as fexinidazole (oral intake) will help to reduce the remaining burden of the disease [3, 4].

Pentamidine (for *gambiense*-HAT) remains the molecule indicated for the treatment of stage 1 of the disease. Thus, stage diagnosis is still necessary. Furthermore, fexinidazole has so far only been used in the context of field trials. What will happen when it is used routinely? Moreover, the infection mechanism remains incompletely understood; the disease may reappear at cyclic intervals as chronically infected individuals without clinical signs of disease or animal reservoirs can lead to reemergence [5, 6]. Recently, the skin has been suggested as possible reservoir of latent infection, leading to relapse [7], and several authors have suggested that relapses can occur from parasites living in the meningeal spaces and recirculating through the cervical lymph node system [8, 9]. As the knowledge of pathogen-invasion mechanisms in the central nervous system (CNS) improves, new concepts help us to integrate data from the field and enhance our knowledge of sleeping sickness infection mechanisms. Establishing disease stages is necessary for effective treatment, and the discovery of new markers in order to do this relies on the understanding of mechanisms of CNS invasion [10].

During stage 1 of the disease, the invasion of the host by the trypanosome is accompanied by inflammation caused by recognition of two pathogen-associated molecular patterns (PAMPs), the parasite VSG coat and DNA, by the immune system [11]. This triggers a cascade of activation of various immune cells, which is regulated by cytokines and chemokines [11–15]. During the course of sleeping sickness, trypanosomes cross the blood–brain barrier and invade the CNS, leading to the second stage of the disease. This tropism of the parasite for CNS induces neuroinflammation and lymphocyte infiltration in the cerebrospinal fluid (CSF). At present, only a lumbar puncture showing cytorachia of greater than 5 cells/*μ*L or evidence of trypanosomes in the CSF allows the diagnosis of stage 2 of the disease [3, 16]. Alternatively, expression levels of various cytokines and chemokines may track inflammatory changes earlier and may be more relevant for the diagnosis as already demonstrated in various inflammatory conditions, including sleeping sickness [17]. Neopterin is a pyrazino-pyrimidine compound that is synthesized by monocytes and macrophages in response to the production of interferon-*γ* by activated T cells. The increase in the level of expression of neopterin therefore follows the increase in macrophage and T cell activation [18]. In fact, increased neopterin levels are seen early in various pathologies, including malignant tumours such as CNS lymphomas [19] and viral infections [20, 21] including HIV [22, 23], justifying its use as a screen to exclude infected blood from blood donors [24, 25]. CXC-chemokine ligand 13 (CXCL-13) is expressed by B cells and dendritic cells and is involved in B cell migration [26]. Perturbation of plasma levels of CXCL-13 is associated with activation of the host's immune response [27], and CXCL-13 is used as an indicator of breast cancer, cutaneous vasculitis, and various lupus-like condition and bacterial infections [28–31]. CSF CXCL-13 levels are clearly associated with neuroborreliosis [32], neurosyphilis concomitant with HIV [33], and multiple sclerosis [34]. Previous studies have shown that increased levels of CXCL-13 and neopterin could be a good diagnostic marker for sleeping sickness and an indicator of the disease stage [35–37]; rapid detection tests (RDTs) based on the neopterin concentration in the CSF are under investigation.

In this study, classical staging criteria, clinical signs, and new potential biomarkers (namely, neopterin and CXCL-13) were reevaluated in patients from a cohort study in Angola [36, 37]. These results are discussed on the base of the immune response and the physiopathological context of the diseases, arguing in favor of an intermediate stage [38].

## 2. Patients and Methods

### 2.1. Ethics Statement

Ethical clearance was obtained from the Angolan Direccao Nacional de Saude Publica, Ministerio da Saude. Written informed consent was received from these individuals prior to enrolment and/or from their parents or guardians for participants below 18 years of age. Any individual who declined to participate was followed up according to the standard procedures of the national control programme of Angola.

The clinical data used to support the findings of this study are restricted by the Angolan Direccao Nacional de Saude Publica, Ministerio da Saude in order to protect patient privacy. Data are available from the authors (bertrand.courtioux@unilim.fr) for researchers who meet the criteria for access to confidential data.

### 2.2. Case Definition, Inclusion, and Exclusion Criteria

**Figure 1:**
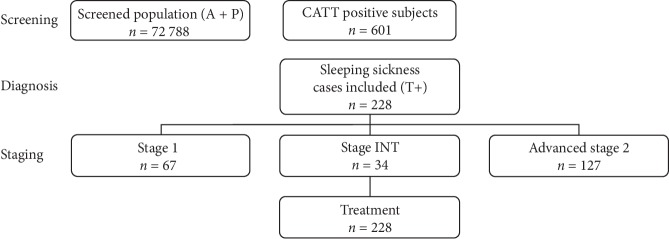
Cohort study conducted in Angola between 2008 and 2011. The enrolment process of 247 *Trypanosoma brucei gambiense-*infected patients and controls is shown. This cohort is composed of 19 controls, 67 patients with stage 1, 34 patients with intermediate stage, and 127 patients with advanced stage 2. All HAT patients were treated according to their stage.

Samples were obtained retrospectively from a cohort study conducted in Angola between 2008 and 2011 (Figure 1). The study aimed to collect appropriate clinical, neurological, psychiatric, and biological data from a cohort of 247 *Trypanosoma brucei gambiense-*infected patients (*n* = 228) from diagnosis to the end of follow-up (6, 12, 18, and 24 months) and noninfected controls (*n* = 19).

It is not acceptable to perform a lumbar puncture on a patient for whom no neurological pathology is suspected. Seronegative Angolan controls for HAT could therefore not be included in this study. Thus, the 19 controls are derived from the 601 patients with a positive CATT test. Initially, these individuals were suspected of HAT and finally considered healthy following further negative tests.

Subjects were enrolled during both active and passive screening activities by teams of the national sleeping sickness control programme. The CATT (Card Agglutination Trypanosomiasis Test) screen was used [39] and followed by confirmation using microscopy of concentrated blood and CSF. Patients with sleeping sickness were defined as individuals in whom trypanosomes were demonstrated in either blood, lymph node aspirate, or CSF by microscopy. Disease cases were classified as stage 1 when no trypanosomes were observed in CSF and when the CSF white blood cell (WBC) count was lower than or equal to 5 cells/*μ*L, whereas those with trypanosomes in the CSF and/or a CSF WBC count above 20 cells/*μ*L were classified as advanced stage 2. Patients who were deemed to be in the intermediate stage were those between stage 1 and advanced stage 2 with a CSF WBC count between 5 and 20 cells/*μ*L and/or with trypanosomes in CSF without an increase in CSF WBC numbers. The Angolan national control programme differentiates the intermediate stage, as they treat those patients with first-stage drugs unless some clinical signs are suggestive of CNS involvement, in which case second-stage drugs are used. All participants were examined clinically, and a questionnaire was used to note all clinical and neurological characteristics (Romberg, Babinski's reflex, dysmetry, meningeal syndrome, etc.), including sleep and psychiatric disturbances using the Mini-International neuropsychiatric interview [40] and the Hamilton rating scale for depression. These two scales include sleep examination criteria. All participants were checked clinically and microscopically for the presence of the main parasitic coinfections (malaria by blood smear, filariasis during blood examination by capillary tube centrifugation, and schistosomiasis when blood was detected in urine samples). The HIV and syphilis status was determined retrospectively on stored samples using the VIKIA HIV 1/2 test (Biomérieux, France) and the RPR-Nosticon II (Biomérieux, France) and TPHA (Biorad, France) tests, respectively. These analyses aimed to exclude individuals with interfering parameters for CSF analysis. Children under 12 years were also excluded to avoid complications in obtaining lumbar puncture samples and also possible difficulties in interpretation [41, 42].

For our analysis of neopterin and CXCL-13, we selected 83 patients for whom we had maximum of neopterin and CXCL-13 CSF concentrations data. When the concentration data were missing and we had CSF samples available in the biobank, we determined the neopterin and CXCL-13 concentration in the CSF via ELISA. The ELISA kits for the dosage of neopterin and CXCL-13 used are the same throughout the study [42].

### 2.3. Sample Collection and Selection

A lymph node aspirate was taken from any subject who presented with swollen lymph nodes and examined for trypanosomes by microscopy. 10 mL venous blood with heparin as anticoagulant was collected from CATT-positive individuals as well as from patients with swollen lymph nodes. 600 *μ*L blood was used to perform capillary tube centrifugation test (4 capillary tubes of 75 *μ*L), and 300 *μ*L blood was used for the miniature anion exchange centrifugation technique [43]. For individuals who were positive by CATT on whole blood, 1 mL plasma was used to perform CATT dilutions. Confirmed cases of parasite infection and/or individuals found positive by CATT at a dilution of 1/16 who had negative results for all other performed tests for parasite infection underwent a lumbar puncture, in accordance with national guidelines for stage determination and/or parasitological confirmation in CSF, when there were suggestive neurological signs. Parasitological examination of CSF was done using the modified single centrifugation technique. This optimized parasitological confirmation method permits sensitivity of parasite detection similar to that of molecular testing [42, 43].

All plasma, buffy coat, and CSF samples that remained after the diagnostic procedures were aliquoted and stored in liquid nitrogen before being transported to Limoges on dry ice and then stored at −80°C. Patient medical data were anonymized. Samples were further selected for neopterin and CXCL-13 determination at diagnosis [42].

### 2.4. Test Procedures

Commercially available ELISA assays were used to measure CSF levels of neopterin (Brahms, Thermo Fisher Scientific, Germany) and CXCL-13 (R&D Systems, UK, and RayBiotech, GA, USA) as performed previously by Tiberti et al. [36, 37]. All assays were performed according to the manufacturer's instructions and the interassay variability was evaluated using quality controls (coefficient of variation (CV) < 20%). A limit of detection corresponding to the mean concentration measured for the lowest standard less than 2 standard deviations was calculated for each assay. All data were obtained using the same ELISA assays than previous authors and data were compared to those obtained by Tiberti et al. [36, 37].

### 2.5. Statistical Analysis

Descriptive analysis was carried out on Microsoft Excel. Receiver operating characteristic (ROC) curves were drawn to determine cut-off points as the best sensitivity/specificity ratio for each marker (CXCL-13, neopterin, leucocyte count, sleep disturbances, neurological signs, and presence of trypanosomes). ROC curves were further used to determine the disease stage (that is, stage 1, intermediate, or advanced stage 2) on diagnostic samples. The significance was assessed using the Kruskal–Wallis test and Fisher's exact test for testing the null of independence (*p* < 0.05). Tests were performed using R Core Team version 3.3.2 [44] and the following packages were used: ROCR for drawing curves and determining the values of specificity and sensitivity that will make it possible to find the cut-off [45] and PMCMR for the pairwise comparisons post hoc test of the Kruskal–Wallis test [46].

**Figure 2:**
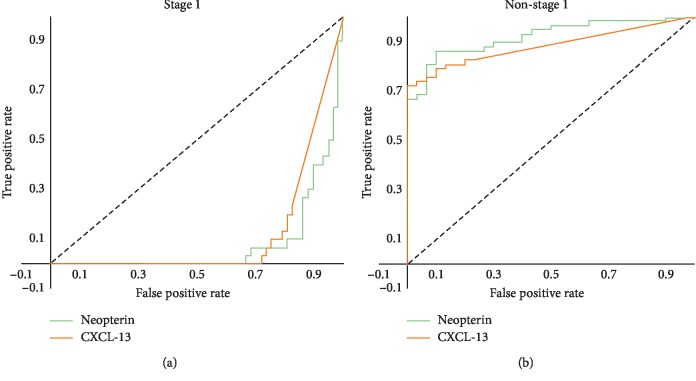
ROC curves for all threshold values for neopterin and CXCL-13 in patients with stage 1 disease and in patients with non-stage 1 disease (intermediate stage and advanced stage 2). ROC curves represent sensitivity as a function of the complement of specificity (1 − specificity) for all threshold values for neopterin and CXCL-13 in patients with stage 1 disease (a) and in patients with non-stage 1 disease (b).

When the values of the CXCL-13 and neopterin biomarkers from the stage 1 patients are represented on the same curve (Figure 2(a)), there is no definite cut-off value with an acceptable sensitivity and specificity. For the feasibility of our analysis, we have thus grouped all patients not in stage 1 (that is patients with intermediate stage and advanced stage 2 disease) and defined a cut-off discriminating non-stage 1 patients (Figure 2(b)). Patients with values below these cut-off points are considered to be in stage 1.

Cut-off points used for comparison have been used in accordance with previous data for neopterin and CXCL-13 [36, 37].

## 3. Results

### 3.1. Population Characteristics at Inclusion

**Figure 3:**
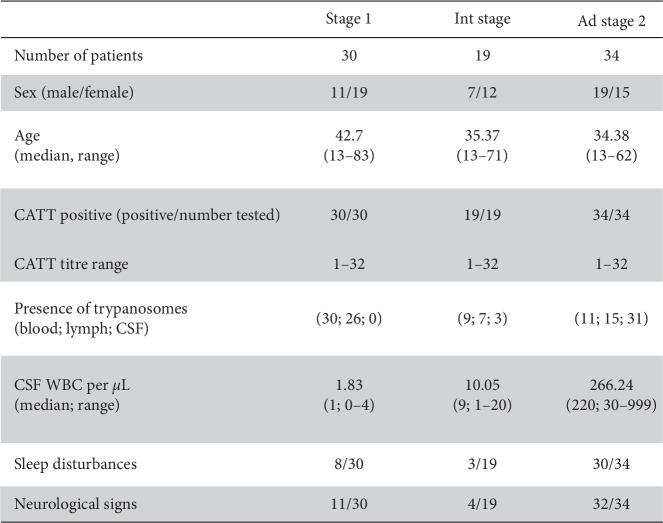
Samples and patients' follow-up with population characteristics at inclusion. Table represents the epidemiological and biological data of the 30 patients with stage 1 disease, the 19 patients with intermediate stage disease, and the 34 patients with advanced (Ad) stage 2 disease who constitute the inclusion cohort.

Demographic, clinica, l and biological data are summarized in Figure 3. Sex ratio was 0.44 (37/83), and ages ranged from 13 to 83, with median ages of 42.7, 35.37, and 34.38 years for stage 1, intermediate, and advanced stage 2 disease groups, respectively. CATT serology was positive in all individuals, as it was the screening method used, and titres ranged from 1/8 to 1/32. Trypanosomes were found in 48 lymph node aspirates, 50 blood samples, and 34 CSF samples. Patients with stage 1 disease had a mean CSF WBC count of 1.83 cells/*μ*L, and no trypanosomes were detected in the CSF. Patients with intermediate stage disease had a mean WBC count of 10.05, and three patients had trypanosomes in their CSF, although the WBC counts of these patients were less than 5 cells/*μ*L. Patients with advanced stage 2 disease had a mean cell count of 226 cells/*μ*L, and trypanosomes were detected in the CSF of 31 out of 34 patients. Sleep disorders were present in 27% of patients with stage 1 disease, 16% of patients with intermediate stage disease, and 88% of patients with advanced stage 2 disease, with a clear increase in their severity with disease stage. Eleven (37%) patients in stage 1 and four (21%) patients in the intermediate stage showed neurological signs, and there was an increase in neurological signs in advanced stage 2 patients (32 out of the 34 (94%)) compared with previous stages (Figure 3). All patients with stage 1 disease were treated with pentamidine; 33 out of 34 patients with advanced stage 2 disease were treated with eflornithine (DFMO), and one was treated with pentamidine; 16 out of 19 patients with intermediate stage disease were treated with pentamidine, and three of them were treated with DFMO. The choice of treatment for intermediate stage patients was made by the medical team based on the severity of the clinical symptoms of these patients.

### 3.2. Neopterin and CXCL-13 Levels during Diagnosis

Neopterin and CXCL-13 concentrations at inclusion are presented in Table 1. At diagnosis, the mean concentrations of neopterin and CXCL-13 in the CSF were higher in patients with advanced stage 2 disease (280.62 nmol/L and 3919.32 pg/mL, respectively) than patients with intermediate disease (18.72 nmol/L and 111.85 pg/mL, respectively) and patients with stage 1 disease (12.15 nmol/L and 26.42 pg/mL, respectively).

### 3.3. New Neopterin and CXCL-13 Cutoff Points for Defining Disease Stages

Our study suggests that a CSF neopterin concentration less than or equal to 15.56 nmol/L defines stage 1 disease with 84% sensitivity and 90% specificity (AUC = 0.91), whereas a concentration above 60.31 nmol/L characterizes advanced stage 2 disease (AUC = 0.99; 97% sensitivity and 100% specificity). Values between these two cut-off points could be used to characterize an intermediate stage (Table 2).

Our findings also suggest that CSF CXCL-13 levels below 91.21 pg/mL define stage 1 disease (AUC = 0.89; 81% sensitivity and 90% specificity), and CXCL-13 levels above 395.45 pg/mL define advanced stage 2 disease (AUC = 0.93; 97% sensitivity and 100% specificity). Values between these two cut-off points could be used to characterize an intermediate stage (Table 2).

### 3.4. Comparison of Neopterin and CXCL-13 Concentrations to Clinical Manifestations (Neurological Signs, Sleep Disorders), Leucocyte Count, and Presence of Trypanosomes in CSF

ROC curves show that neurological signs and sleep disorders appeared when CSF neopterin concentrations were above 21.20 nmol/L (AUC = 0.79 and 0.81, respectively) and/or CXCL-13 concentrations were above 330.26 pg/mL (AUC = 0.78 and 0.80, respectively). Trypanosomes were found in the CSF when neopterin concentrations were higher than 31.40 nmol/L (AUC = 0.94) and/or CXCL-13 concentrations were higher than 688.85 pg/mL (AUC = 0.94). Sleep and neurological disorders were associated with the presence of a minimum of 16 cells/*μ*L of CSF (AUC = 0.80), and the invasion of the CNS by trypanosomes was associated with the presence of more than 50 cells/*μ*L of CSF (AUC = 0.93). The sensitivity and specificity of each of these cut-off points are shown in Table 3.

## 4. Discussion

Our study addressed and characterized a specific group of patients with intermediate stage sleeping sickness, or early stage of CNS involvement. There have been reports of patients cured with first-line drugs at this disease stage, which suggests that either pentamidine [38, 47, 48] or host immunity can contribute to eradication of the parasite [49, 50]. Our results showed that 75% (12/16) of patients with intermediate stage disease were cured following pentamidine treatment, confirming earlier data and supporting evidence of the existence of that stage [38, 51]. The physiopathology of this intermediate stage remains unknown. Question remains about the presence and the numbers of parasites in the CNS, the host immune response. Some hypothesis can be done, especially about the capacity of the parasite to resist in the CNS but its localisations into cells were demonstrated *in vitro* [52, 53]. In the present state of knowledge, this intermediate stage (between the hemolymphatic stage and the meningoencephalitis stage) can be defined only on the basis of our biomarker levels CXCL-13 and neopterin. But we can hypothesized that it corresponds to the presence of the parasite into cells as it was shown previously *in vitro* and/or the beginning of the BBB crossing with the presence of some parasites in the CSF which have not been detected yet on CSF after lumbar puncture. However, the relevance and the importance of the intermediate stage are still not completely understood [10]. In three patients with intermediate stage disease, trypanosomes were present in the CSF or at the interface between the blood system and the CSF which are endothelial cells, without an increase in WBC count. Current WHO recommendations for clinical trials do not usually take the intermediate stage of disease into account in order to simplify treatment protocols; therefore, stage 2 disease is characterized as having a CSF WBC of above 5 cells/*μ*L [52]. In the next years, those criteria will not be necessary for treatment, as fexinidazole will be proposed for all patients. But the accuracy of using neopterin and CXCL-13 as biomarkers for sleeping sickness has been demonstrated previously in larger cohorts, and their links with early inflammatory processes of sleeping sickness have been shown [35–37]. In our study, we assessed the ability of those markers to potentially characterize disease stages; it was shown that levels of CSF neopterin and CXCL-13 can define stage 1 and advanced stage 2 diseases and that the range of biomarker concentrations between those assigned to stage 1 and advanced stage 2 could indicate an intermediate disease stage.

One of the difficulties encountered was to define cut-off points for markers of stage 1 disease in the absence of seronegative Angolan controls for HAT in our cohort. This is why we have grouped all patients in the intermediate stage and in stage 2 of the disease and therefore defined cut-offs to discriminate patients who are not in stage 1 (non-stage 1). Values below these thresholds are therefore characteristic of stage 1 patients.

Previously, CSF neopterin levels below 14.3 nmol/L were indicative of stage 1 disease, and neopterin levels greater than this were indicative of stage 2 disease (determined from 412 samples) [37]. Similarly, CSF concentrations of CXCL-13 lower than 125.5 pg/mL were indicative of stage 1 disease, whereas levels greater than this were indicative of stage 2 disease (determined from 97 samples) [36]. In our study, it was shown that the cut-off point of CSF neopterin to define stage 1 disease is below 15.56 nmol/L, which is close to that established by Tiberti et al. in 2013 [37]. However, we have established a cut-off point marking the entry of patients in advanced stage 2 at 60.31 nmol/L, introducing a gap between two cut-off values that represents the levels of neopterin found in the intermediate stage of disease.

In our study, the cut-off point of CSF CXCL-13 to define stage 1 disease was found to be a concentration of less than 91.21 pg/mL; a CSF CXCL-13 concentration greater than 395.45 pg/mL was indicative of advanced stage 2 disease. Concentrations of CXCL-13 between these two values were indicative of the intermediate stage. The concentrations of CXCL-13 that define stage 1 and stage 2 diseases were originally found in the study by Tiberti et al. in 2012 [36]; their data suggested that a CSF CXCL-13 concentration below 125.5 pg/mL was indicative of stage 1 disease, whereas values above this threshold were indicative of stage 2 disease. This cut-off value falls between the two cut-off points found in our study (below 91.21 pg/mL for stage 1 disease and above 395.45 pg/mL for advanced stage 2).

This observed difference between the CXCL-13 and neopterin cut-off points is due to the fact that in the studies by Tiberti et al. [36, 37], patients in intermediate stages of disease are included in stage 2. Here, we have demonstrated that a change in the CSF neopterin and CXCL-13 concentration does not correspond simply to stage 1 or 2 (a WBC count of greater than 5 cells/*μ*L), but rather to an intermediate stage between stage 1 (a WBC count below 5 cells/*μ*L) and an advanced stage 2 (a WBC count greater than 20 cells/*μ*L).

The differences in levels of neopterin and CXCL-13 in patients with sleeping sickness may be explained by their production mechanisms. Neopterin is produced by microglial cells in response to the parasite [54, 55], whereas CXCL-13 occurs when the parasite is already in the CNS and induces, in conjunction with CXCL-12, leucocyte extravasation into the CSF [56]. This is correlated with a late response of the B cell populations, as described in the literature [57, 58]. This mechanism can explain why, in serum, no correlation can be made with stage diseases.

The main criticism of this work is the absence of a HAT-seronegative Angolan controls population, which is justified because it is not ethical to perform a lumbar puncture on a healthy person. It is for this reason that we had to define cut-off points for neopterin and CXCL-13 above which patients are considered non-stage 1; all patients with values below these thresholds are thus stage 1. According to the literature, the normal concentration of neopterin is less than 5.1 nmol/L of CSF [19], and the normal concentration of CXCL-13 is less than 11.5 pg/mL of CSF [59], but we do not know the true concentration values for these two biomarkers in healthy Angolan individuals. In our study, cut-off values (as well as their sensitivities and specificities) were defined on a limited sample size. These values would be improved using a larger sample of patients.

The difficulty in all studies of neurological stage discrimination during sleeping sickness is the definition of the gold standard as recently reviewed [60]. The current gold standard is defined by the criteria of the WHO [54] based on the cytorachia and/or the presence of the parasite in the CSF. The only specific test confirming stage 2 is the presence of trypanosomes in the CSF (neurological invasion) [61]. In our study, the presence of trypanosomes is a good marker when CSF WBC count is over 50 cells/*μ*L of CSF (91% sensitivity and 96% specificity) and confirms the lack of sensitivity of parasite detection. The most sensitive and current test to define CNS involvement is cytorachia but this test lacks specificity [54, 62]. The onset of neurological signs and sleep disorders may be associated with cytorachia of less than 5 cells/*μ*L; this has been reported previously [63, 64], confirmed in our study with 37% of stage one patients with neurological signs, and shown also more recently in T. b. *rhodesiense* infection where patients are cured with first-stage drugs [16]. Thus, the appearance of WBC in the CSF does not always correlate with the appearance of neurological signs and sleep disorders; this would also suggest that cytorachia is not a reliable biomarker for staging. In our study, neurological signs and sleep disorders appeared when mean cytorachia was greater than 16 cells/*μ*L. We established cut-off points for neopterin and CXCL-13 related to the onset of clinical signs of the disease instead of using cytorachia but the neopterin and CXCL-13 cutsoff points were not discriminative enough to be used as diagnostic markers.

## 5. Conclusion

Our work suggests that the levels of the two biomarkers, neopterin and CXCL-13, are good markers of disease staging, in the patient. Using the two biomarkers in conjunction, rather than individually, could be a stronger predictor of disease stage after its diagnosis.

Biomarker identification should not be limited to the CSF and should be continued on other biological fluids to avoid lumbar puncture. Even if no correlations were observed in serum, neopterin and CXCL-13 must be in urine and lacrimal fluid. With the development of new methods to identify specific markers, as proteomics, diagnosis algorithms should be simplified in the near future.

## Figures and Tables

**Table 1:** CXCL-13 and neopterin levels in the three stages of disease.

		Inclusion
		Mean (SD)
Stage 1	CXCL-13^1^	26.42 (36.38)
	Neopterin^2^	12.15 (4.95)

Int stage	CXCL-13^1^	111.85 (119.30)
	Neopterin^2^	18.72 (8.5)

Ad stage 2	CXCL-13^1^	3919.32 (1856.06)
	Neopterin^2^	280.62 (220.69)

The mean and standard deviation (SD) of CSF concentration of neopterin and CXCL-13 are shown for patients with stage 1, intermediate (Int), and advanced (Ad) stage 2 disease at inclusion. ^1^pg/mL. ^2^nmol/L.

**Table 2:** Sensitivity and specificity of new tests for staging.

	Neopterin (nmol/L)	CXCL-13 (pg/mL)
Non-stage 1	15.57	91.21
Sensitivity [IC]	0.90 [0.73; 0.98]	0.90 [0.73; 0.98]
Specificity [IC]	0.86 [0.74; 0.94]	0.79 [0.66; 0.89]

Int stage		
Sensitivity [IC]	0.65 [0.43; 0.84]	0.52 [0.31; 0.73]
Specificity [IC]	0.94 [0.85; 0.98]	0.95 [0.87; 0.99]

Ad stage 2	60.31	395.45
Sensitivity [IC]	0.97 [0.85; 1.00]	0.90 [0.85; 1.00]
Specificity [IC]	1.00 [0.93; 1.00]	0.86 [0.93; 1.00]

The cut-off values established for neopterin and CXCL-13 are expressed in nmol/L and pg/mL, respectively. The sensitivity and specificity values for each cut-off points determined by ROC analysis are shown in the table. The authors defined cut-off values of neopterin and CXCL-13 for non-stage 1 patients, under which patients are considered to be in stage 1 of sleeping sickness.

**Table 3:** Comparison of neopterin and CXCL-13 results to classical markers (quantitative and qualitative data).

Cut-off	Neurological signs	Sleep disorders	Trypanosomes in CSF
CXCL-13^1^	310.52	330.26	310.52
Sensitivity [IC]	0.63 [0.49; 0.76]	0.63 [0.48; 0.77]	0.79 [0.63; 0.90]
Specificity [IC]	0.92 [0.77; 0.99]	0.92 [0.78; 0.98]	0.90 [0.78; 0.97]
Neopterin^2^	21.20	21.20	31.41
Sensitivity [IC]	0.69 [0.54; 0.81]	0.69 [0.55; 0.82]	0.84 [0.69; 0.94]
Specificity [IC]	0.83 [0.67; 0.94]	0.82 [0.68; 0.92]	0.90 [0.78; 0.97]
WBC count^3^	12	16	50
Sensitivity [IC]	0.71 [0.56; 0.83]	0.71 [0.57; 0.83]	0.82 [0.66; 0.92]
Specificity [IC]	0.92 [0.78; 0.98]	0.89 [0.76; 0.97]	0.84 [0.70; 0.93]

The determined cut-off values of neopterin, CXCL-13, and leucocyte numbers at the appearance of clinical signs of the disease (neurological signs and sleep disorders) and CNS invasion by the parasite. ^1^pg/mL. ^2^nmol/L. ^3^cells/*μ*L CSF.

## Data Availability

The clinical data used to support the findings of this study are restricted by the Angolan Direccao Nacional de Saude Publica, Ministerio da Saude, in order to protect patient privacy. Data are available from the authors (bertrand.courtioux@unilim.fr) for researchers who meet the criteria for access to confidential data.
